# Fish Cytogenetics: Present and Future

**DOI:** 10.3390/genes12070983

**Published:** 2021-06-28

**Authors:** Anna Rita Rossi

**Affiliations:** Dipartimento di Biologia e Biotecnologie “C. Darwin”, Sapienza-Università di Roma, Via Alfonso Borelli 50, 00161 Rome, Italy; annarita.rossi@uniroma1.it; Tel.: +39-0649918014

Fish is the most species-rich class of vertebrates, including a number of species that correspond to about half of the total vertebrates [1]. This diversity is the result of more than 500 million years of evolution, which allowed fish to exploit niches in all aquatic ecosystems, in association with the geological and climatic transformation that affected seas, lagoons, lakes and river basins. Presently, 35,768 valid species are reported in the Eschmeyer fish catalogue [2], and new ones are discovered yearly, mainly from the tropical and subtropical areas [1].

The study of the evolutionary biology of fish took advantage of the application of molecular tools: classical sequence analysis, next-generation technology and approaches of evolutionary developmental biology are recognized within the toolbox of modern fish systematics [1]. In this picture, cytogenetics analysis is dismissed, although karyotype data both represent the pre-requisite for genome analysis and might provide support in demonstrating the existence of reproductive barriers. As an example, in the last decade, new marine and freshwaters sibling (or cryptic) species and parapatric divergent populations were disclosed thanks to cytogenetic analysis [3,4,5,6]. These cases represent exceptions, as the karyotypes (and the genomes) of fishes are poorly studied compared to other vertebrates, or at least not proportionally to the species richness of this group. The last comprehensive review of fish cytogenetic data [7] reported karyotype information derived from traditional staining techniques on 3425 species corresponding to 53 orders and 264 families, that covered about 12.2% of extant fish species (a total of 27,977 known at the date of publication), mainly from freshwaters. Since then, the number of analyzed species increased, and there was an exponential growth of data from molecular cytogenetics through chromosome painting and chromosome mapping of whole genomic DNA or repeated sequences [8]. These approaches opened new scenarios in fish cytogenetics, allowing for in-depth comparisons between related taxa. Meanwhile, the karyotype of many species is still undescribed due to the difficulty in catching samples (a reason that could explain why freshwater fishes are much more represented than marine fishes), in the necessity to have living materials (specimens or cells), in troubling to obtain karyotypes from cell-culture and lastly in the reduced probability of success in obtaining good chromosome figures compared to warmblood vertebrates.

In the last two decades, investigations on fish karyotypes have been integrated with those from genome sequencing: data demonstrated genome plasticity and variability higher than that observed in other vertebrates and provided the opportunity to investigate this taxon from a different perspective. Indeed, recently, the comparative analysis of fish genomes has been used to quantify the rates of divergence based on chromosome number and repetitive sequences distribution vs. single copy sequences [9], for identifying sex determination genes and reconstructing gene family evolution in different teleost species [10], or to gain information on the diversification of fish groups [11]. Presently, the genomes of more than 80 fish species are annotated in the Ensembl database (https://www.ensembl.org/index.html, accessed on 6 April 2021), and about 150 are assembled to the chromosome level at the NCBI webpage (https://www.ncbi.nlm.nih.gov/genome/browse#!/eukaryotes/, accessed on 6 April 2021). However, the quality of most of these assembled genomes (including those of other vertebrates and eukaryotes) has been subject to criticism, as it does not meet the standard metrics proposed for reference genomes [12]. Anyway, the number of fish genomes sequenced is going to increase quickly by 2028, at least for the contribution of the Earth BioGenome Project [12].

The sequencing of the first fish genome (and second vertebrate, after human) dates back to about twenty years ago [13]: the teleost *Takifugu rubripes*, commonly known as the Japanese pufferfish, was chosen as the target species, because of its compact genome (0.39 Gb), one of the smallest in size within vertebrates. Indeed, fishes are known to exhibit genomes that are very plastic and variable in size (from 0.35 to 133 Gb), with Teleost showing the lower values of the range, namely the most compact genomes (0.35–10 Gb) [14]. The large differences in fish genome sizes are associated with the variable amount of repetitive genetic elements [15,16]: their contribution to the total genome is higher than in mammals, and these sequences play a role in divergence and rapid evolution of sex-determining loci [16,17,18]. A high number of variable transposable elements (TEs) is also present within the most compact fish genomes that include a small fraction of repetitive sequences: indeed TEs seem to have a rapid turnover in teleost fish genomes [16].

This issue provides an overview of what is going on in fish cytogenetics based both on review articles on specific taxonomic families, and original research articles, showing the different application of cytogenetic approaches in evolutionary biology and conservation biology. The analyzed species are representatives of a large variety of habitats, from endemic fishes that inhabit remote geographic areas to widespread species used for fisheries and aquaculture. The baseline data available for these taxa were, thus, quite different: for some species (or even genus), the karyotype was unknown, while others belong to well-studied taxonomic groups for which data on repetitive DNA families and genomics were already available. Thus, articles span from traditional cytogenetics to more modern cytogenomics and genome analysis, demonstrating the utility and potential of the application of modern techniques for the improvement of new insight within this field.

The classical cytogenetic papers report the presence of supernumerary chromosomes and/or the mapping of repeated sequences. As an example, one paper focused on the freshwater fish *Dormitator latifrons* that represents an important food resource in Central South America and whose karyotype was still undescribed. In this species, the presence of an XY chromosome system and mapping of repeated sequences repeats allowed the identification of the main mechanisms of chromosome rearrangement that have driven karyotypic evolution in the genus [19]. Other papers reported polymorphism related to the presence of B chromosomes [20,21]. This is the case of the common nase *Chondrostoma nasus*, which belongs to Leuciscidae, a freshwater family whose species are usually characterized by a stable diploid number. Despite this, two common nase populations from different geographic areas showed mitotically unstable submetacentric B chromosomes [20]. Similarly, in the marine ice cod *Arctogadus glacialis* endemic to the Arctic Sea, the cytogenetic analysis revealed a remarkable intraspecific chromosome polymorphism: six karyotype variants associated with the presence of B chromosomes along a latitudinal cline, and different patterns of heterochromatin and rDNA distribution. These karyotype variations can be associated with the presence of isolated fjord populations at different latitudes/environmental condition, raising concerns on the fate of such populations in the light of the ongoing climate-driven environmental changes [21]. Finally, chromosome mapping of major and minor ribosomal gene clusters was applied in a comparative cytogenetic analysis of Muraeinidae, an ancient and poorly studied family. Results unveiled a pattern of ribosomal sequence distribution in contrast to what was generally reported for fishes, showing high variability in the number and location of 5S rDNA clusters, and a tendency to the conservation of the number and location of the 45S rDNA clusters among species [22].

In addition to standard analysis, comparative genomic hybridization was applied in the study of the Amazonian catfish. In details, the investigation of new species and the integration of data from literature showed an extensive variability of the chromosome number in species of the genus *Harttia* and the occurrence of different sex chromosome systems. These features make *Harttia* a suitable model to study sex chromosome evolution in fishes [23].

A fair number of articles focused on species that are of economic interest in fishery/aquaculture, considering hybrids or different developing stages. This is the case of the hybrids obtained from the cross of Russian sturgeon (*Acipenser gueldenstaedtii*) and American paddlefish (*Polyodon spathula*) that allow investigating on the genome duplication and its consequences on the ploidy level in the hybrid progenies, showing the presence of chromosome complements that correspond to “functional” triploids and “functional” pentaploids [24]. There is also the case of the Senegal sole *Solea senegalensis*, for which a comprehensive integrated genetic map of the complete karyotype was presented. These data on the genome organization support the existence of a sex proto-chromosome pair and show that Robertsonian fusions and chromosomal inversions drive the evolution of the karyotype of this species, demonstrating the existence of conserved gene linkages shared with other fish model species [25]. Other examples concern Salmonid species. In detail, in the rainbow trout (*Oncorhynchus mykiss*), quantitative fluorescence in situ hybridization was used to assess the dynamic of telomeres at different developmental stages, and in fertile diploid individuals and sterile triploid specimens that are used in the commercial production [26]. In the Anatolian endemic flathead trout, *Salmo platycephalus*, the classical description of the karyotype was associated with a cytogenomic approach, that allowed the production of a prototypical virtual karyotype of *Salmo trutta* starting from high-quality genome data. This opens future perspectives not only for salmonid, but also for all vertebrates’ cytogenetics [27]. Finally, two papers used the cytogenomic approach, providing new perspectives on genome evolution in vertebrates. The first one investigates at a fine scale AT/GC organization in fish genomes and understands the contribution of repeats to the total GC% [28]. As GC% is associated with gene density and chromatin structure, the results could explain the inability to produce G-banding with traditional cytogenetic methods: in some species, it might provide an alternative occasional banding pattern along the chromosomes. The last paper compares chromosome size, genome size, GC% of repeats and proportion of repeats across fishes and with other vertebrates [29]. This quantitative approach showed that GC% of repeats and proportion of repeats are independent of chromosome size and disclosed an immense diversity in fish, spanning from the enrichment of GC% in the compact genomes observed in modern lineages to lower enrichment of GC% in the larger genomes present in basal fish lineages (with micro-chromosomes). Different strategies/evolutionary paths are observed in the other vertebrates.

Last, but not least, this Special Issue is dedicated to the memory of Prof. Luciana Sola, who was a pioneer in fish cytogenetics and dedicated her entire academic life to this field at the University of Rome Sapienza. Unfortunately, she left us during 2020, the period in which this Special Issue took shape. Many of the people (the younger ones) who contributed to this article collection started their scientific work by reading the articles she authored; others (the older ones) over the years have collaborated with her, exchanging scientific opinions and comments and laughter during the meetings. I had the honour of working with her as a master student and a colleague. All we “fish chromosome people”, will remember her as a special friend.
